# Machine learning cardiac-MRI features predict mortality in newly diagnosed pulmonary arterial hypertension

**DOI:** 10.1093/ehjdh/ztac022

**Published:** 2022-05-02

**Authors:** Samer Alabed, Johanna Uthoff, Shuo Zhou, Pankaj Garg, Krit Dwivedi, Faisal Alandejani, Rebecca Gosling, Lawrence Schobs, Martin Brook, Yousef Shahin, Dave Capener, Christopher S Johns, Jim M Wild, Alexander M K Rothman, Rob J van der Geest, Robin Condliffe, David G Kiely, Haiping Lu, Andrew J Swift

**Affiliations:** Department of Infection, Immunity and Cardiovascular Disease, University of Sheffield, Sheffield, UK; Department of Clinical Radiology, Sheffield Teaching Hospitals, Sheffield, UK; INSIGNEO, Institute for in silico medicine, University of Sheffield, UK; Department of Computer Science, University of Sheffield, Sheffield, UK; Department of Computer Science, University of Sheffield, Sheffield, UK; Department of Infection, Immunity and Cardiovascular Disease, University of Sheffield, Sheffield, UK; Department of Infection, Immunity and Cardiovascular Disease, University of Sheffield, Sheffield, UK; Department of Clinical Radiology, Sheffield Teaching Hospitals, Sheffield, UK; Department of Infection, Immunity and Cardiovascular Disease, University of Sheffield, Sheffield, UK; Department of Infection, Immunity and Cardiovascular Disease, University of Sheffield, Sheffield, UK; Department of Computer Science, University of Sheffield, Sheffield, UK; Department of Infection, Immunity and Cardiovascular Disease, University of Sheffield, Sheffield, UK; Department of Infection, Immunity and Cardiovascular Disease, University of Sheffield, Sheffield, UK; Department of Infection, Immunity and Cardiovascular Disease, University of Sheffield, Sheffield, UK; Department of Infection, Immunity and Cardiovascular Disease, University of Sheffield, Sheffield, UK; Department of Clinical Radiology, Sheffield Teaching Hospitals, Sheffield, UK; Department of Infection, Immunity and Cardiovascular Disease, University of Sheffield, Sheffield, UK; INSIGNEO, Institute for in silico medicine, University of Sheffield, UK; Department of Infection, Immunity and Cardiovascular Disease, University of Sheffield, Sheffield, UK; Leiden University Medical Center, Leiden, The Netherlands; Department of Infection, Immunity and Cardiovascular Disease, University of Sheffield, Sheffield, UK; Sheffield Pulmonary Vascular Disease Unit, Royal Hallamshire Hospital, Sheffield, UK; Department of Infection, Immunity and Cardiovascular Disease, University of Sheffield, Sheffield, UK; INSIGNEO, Institute for in silico medicine, University of Sheffield, UK; Sheffield Pulmonary Vascular Disease Unit, Royal Hallamshire Hospital, Sheffield, UK; INSIGNEO, Institute for in silico medicine, University of Sheffield, UK; Department of Computer Science, University of Sheffield, Sheffield, UK; Department of Infection, Immunity and Cardiovascular Disease, University of Sheffield, Sheffield, UK; Department of Clinical Radiology, Sheffield Teaching Hospitals, Sheffield, UK; INSIGNEO, Institute for in silico medicine, University of Sheffield, UK

**Keywords:** Machine learning, Artificial Intelligence, Cardiac MRI, Prognosis, Mortality, Pulmonary hypertension

## Abstract

**Aims:**

Pulmonary arterial hypertension (PAH) is a rare but serious disease associated with high mortality if left untreated. This study aims to assess the prognostic cardiac magnetic resonance (CMR) features in PAH using machine learning.

**Methods and results:**

Seven hundred and twenty-three consecutive treatment-naive PAH patients were identified from the ASPIRE registry; 516 were included in the training, and 207 in the validation cohort. A multilinear principal component analysis (MPCA)-based machine learning approach was used to extract mortality and survival features throughout the cardiac cycle. The features were overlaid on the original imaging using thresholding and clustering of high- and low-risk of mortality prediction values. The 1-year mortality rate in the validation cohort was 10%. Univariable Cox regression analysis of the combined short-axis and four-chamber MPCA-based predictions was statistically significant (hazard ratios: 2.1, 95% CI: 1.3, 3.4, *c*-index = 0.70, *P* = 0.002). The MPCA features improved the 1-year mortality prediction of REVEAL from *c*-index = 0.71 to 0.76 (*P* ≤ 0.001). Abnormalities in the end-systolic interventricular septum and end-diastolic left ventricle indicated the highest risk of mortality.

**Conclusion:**

The MPCA-based machine learning is an explainable time-resolved approach that allows visualization of prognostic cardiac features throughout the cardiac cycle at the population level, making this approach transparent and clinically interpretable. In addition, the added prognostic value over the REVEAL risk score and CMR volumetric measurements allows for a more accurate prediction of 1-year mortality risk in PAH.

## Graphical Abstract

**Graphical abstract:**
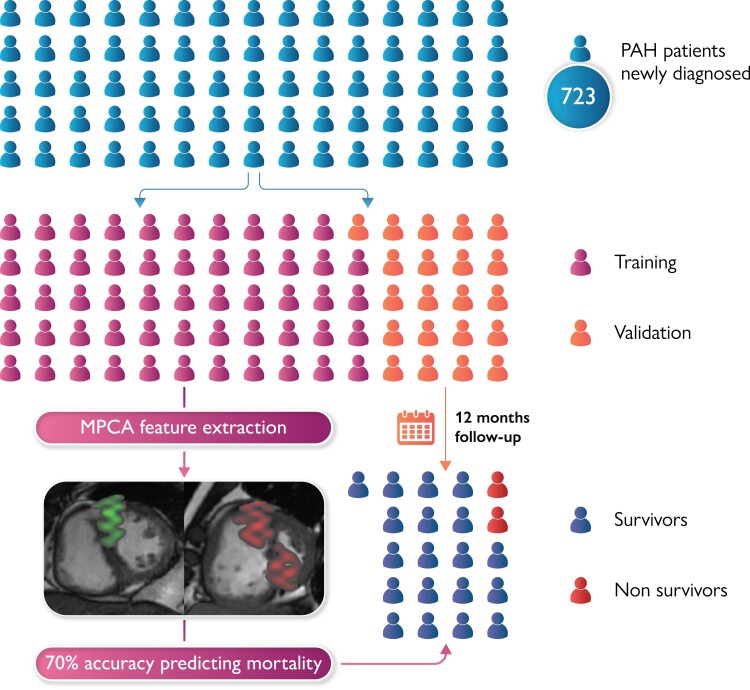
Prognostic features on CMR can be extracted using multilinear principal component analysis (MPCA) machine learning to predict outcome in pulmonary arterial hypertension (PAH).

## Introduction

Cardiac magnetic resonance (CMR) is a powerful prognostic tool owing to its ability to assess cardio-physiological parameters such as the volume and function of the cardiac chambers, tissue characterization, and anatomical structure. Machine learning methods harnessing CMR’s prognostic abilities remain rare and mainly focus on segmenting cardiac chambers to automate CMR measurements.^1^ The process of automating CMR measurements has matured over recent years, proving to be accurate and comparable with results obtained from manual segmentation.^2–4^ However, there is a wealth of data available in CMR studies other than those based on volumetric measurements. A recent machine learning model based on the motion of segmented right ventricle predicted mortality in a mixed cohort of pulmonary hypertension patients.^5^ This study linked impaired basal longitudinal shortening and transverse contraction at the interventricular septum and free wall with an increased risk of mortality.^5^ Another recent machine learning model based on CMR disease features extracted by multilinear principal component analysis (MPCA) has been used to predict the presence or absence of pulmonary arterial hypertension (PAH)^6^ without the need for segmentation.

The MPCA-based model is interpretable because MPCA is a linear and transparent feature extraction method, thus a particularly promising machine learning approach for CMR imaging. Each CMR image sequence is a three-dimensional array (e.g. 512 × 512pixels × 6 mm slice thickness ×20images throughout the cardiac cycle), with each element being a voxel capturing different tissue characteristics, anatomical location, and temporal variation in the cardiac cycle. Such a multidimensional array can be naturally represented as a mathematical object called a tensor. MPCA extracts features directly from such multidimensional tensor representation which preserves the multidimensional structure of the original CMR data more accurately than reshaping it into one-dimensional, vector representation.^7^ The extracted MPCA features can then be weighted in classification or regression models to optimize the prediction of the desired outcome.

Pulmonary arterial hypertension is a rare but serious disease that is associated with high mortality if left untreated.^8^ This study aims to assess the prognostic accuracy of the above MPCA-based model to predict 1-year mortality in PAH. Therefore, evaluating prognosis is key to identifying high-risk patients and optimizing their management strategies as recommended by the European Society of Cardiology guidelines.^9,10^ Multiple clinical parameters are routinely obtained to evaluate PAH disease progression, including pulmonary haemodynamics from right heart catheterization (RHC), functional data from exercise tolerance and pulmonary function tests, biochemistry including N-terminal pro-B-type natriuretic peptide (NT-proBNP) and imaging including echocardiogram and CMR. The REVEAL score is a composite clinical risk score for mortality that combines these clinical parameters to predict 1-year mortality.^11^ In addition, CMR measurements such as right ventricular volumes and function have been shown to predict mortality in PAH.^12^ Thus, the availability of detailed patient phenotyping and prediction scores allows setting a clinical benchmark for the performance of machine learning prognostic models in PAH. This study assesses the additive value of the MPCA-based model to predict mortality compared with established prognostic parameters such as the REVEAL risk score and CMR measurements.

## Methods

The TRIPOD checklist for reporting prediction model development and validation was followed^13^ and is available in the supplemental material.

### Study population

All consecutive treatment-naïve patients with PAH referred for a baseline CMR between 2008 and 2019 were identified from the ASPIRE registry.^14^ Eligibility criteria included: (i) a baseline CMR study performed within 14 days of a PAH diagnosis, confirmed by RHC, and before the commencement of PAH treatment. (ii) Minimum 12 months follow-up or death within 12 months post-CMR study. The study population was divided into two cohorts: (i) a training cohort whose CMR images were used to develop and optimize the prognostic algorithm and (ii) a validation cohort that was left out of training and used to validate the performance of the prognostic model. The cohort was split 70:30 into the model development and model validation cohort.

Ethical approval was obtained from the local ethics committee and written consent was waived for this retrospective study (ref c06/Q2308/8).

### MR imaging protocol

Cardiac magnetic resonance was performed with a 1.5 Tesla GE HDx (GE Healthcare, Milwaukee, USA) system using an eight-channel cardiac coil. Four-chamber (4Ch) and short-axis (SA) cine images were acquired using a cardiac-gated multislice balanced steady-state free precession sequence (20 frames per cardiac cycle, slice thickness 10 mm, 0 mm inter-slice gap, field of view 480 mm, acquisition matrix256 × 200, flip angle 60°, BW 125 KHz/pixel, TR/TE 3.7/1.6 ms). A stack of images in the SA plane were acquired fully covering both ventricles from base to apex. End-systole was considered to be the smallest cavity area. End-diastole was defined as the first cine phase of the R-wave triggered acquisition or largest volume. Patients were in the supine position with a surface coil and with retrospective ECG gating.

Volumetric and ventricular function analysis was performed by contouring the ventricular endocardial borders at end-diastole and end-systole on the SA images using MASS software (MASS, 2020; Leiden University Medical Center, Leiden, the Netherlands). Papillary muscles and trabecula were included in the blood volume.

### Image preprocessing

**Figure 1:**
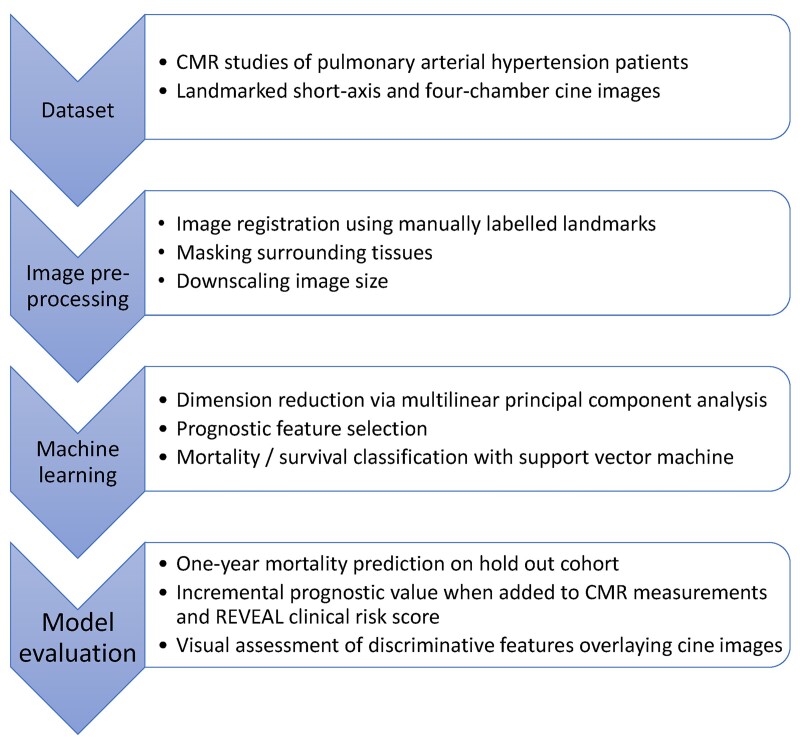
Model pipeline flow chart.

Mid-chamber SA and 4Ch cine images were used in this study. Images were processed following methods in a previous study.^15^ In brief, images were preprocessed by standardizing CMR voxel units between subjects, registering to each other using three anatomical landmarks, masking surrounding tissues, and downscaling image size (*Figure 1*).

Cardiac magnetic resonance voxel units were standardized between subjects by *z*-scores. Rigid image registration was used based on three predefined fixed anatomic landmarks. The landmarks were manually placed on SA (superior insertion point; right ventricular free wall inflexion; mid-left ventricular lateral wall) and 4Ch (left ventricle apex; lateral mitral annulus; lateral tricuspid annulus). Cardiac magnetic resonance images were landmarked by a single reader (S.A.) with independent visual quality assurance checks (S.A.; J.U.). To focus on spatially relevant features, an ellipsoidal mask was fitted around the heart. Downsampling was performed to four image sizes (32 × 32, 64 × 64, 128 × 128, and 256 × 256).

### Multilinear principal component analysis pipeline

The prognostic prediction was achieved by training support vector machines (SVMs) on MPCA features extracted from CMR studies.^6,7^ The methodology followed the MPCA-based pipeline in previous studies.^6,15^ This pipeline was trained through 10 rounds of 10-fold cross-validation on the development cohort (*n* = 516). For each fold during training, MPCA features were extracted and ranked for prognostic capability using Fisher’s Discriminant Analysis in the following way. Extracted features were ranked and selected using a step-wise feature inclusion method. This was performed using a random tuning-set (*n*≈50) of cases. The feature set with the highest tuning-set performance was used to train an SVM and tested on the left-out fold. The feature set with the highest fold-performance was used to train the final development SVM. This MPCA-based machine learning model was then applied to the completely left-out validation cohort (*n* = 207). On a standard computer, the time it takes to process each image and perform inference is much <0.1 second. A Jupyter notebook tutorial of the open-source pipeline code is available at: https://colab.research.google.com/github/pykale/pykale/blob/main/examples/cmri_mpca/tutorial.ipynb

### Visualization of tensor features

Trained features were visualized by using MPCA reconstruction to obtain spatially relevant feature maps. To visually inspect the impact of specific regions on high- and low-risk of mortality prediction, a two-step procedure of thresholding and clustering was implemented. Voxels containing high absolute values (high positive = high-risk, high negative = low-risk) of MPCA features were thresholded. Morphological dilation-erosion using a spherical structural element (*r* = 2) was performed and clusters of visually significant size were overlaid on individual patients’ original CMR scans.

### Clinical and mortality data

Clinical data including intermittent shuttle walking test, pulmonary function test, and serum level of NT-proBNP were collected before treatment was commenced. Demographic data, WHO functional status, PAH subgroup diagnosis, and outcome were collected from the electronic medical system.Mortality data were collected from the electronic records of the National Health Service (NHS) Personal Demographics Service. The NHS automatically updates the mortality records once a death is registered in the United Kingdom. All patients were followed up as part of the national service specification for patients with pulmonary hypertension for a minimum of 12 months. No patients were lost to follow-up.

### Statistical analysis

Continuous variables are presented as proportions, means ± standard deviations, or median and interquartile range for data not following a normal distribution. The sample size for developing the prediction model was calculated using a 1-year mortality prevalence of 10% and seven predictor parameters and required 420 patients to develop the mortality prediction model.^16^ The REVEAL score was calculated from composite clinical parameters^11^ and modified to include the incremental shuttle walk test instead of the 6 min walking test.^17,18^ The CMR volumetric measurements were indexed for body surface area and corrected for age and sex by calculating the percentage predicted values as per published reference data.^19,20^ The outcome of the MPCA-based pipeline was calculated as the SA and 4Ch probabilities based on the SVM prediction. A combined probability was calculated by further training a dual-scan SVM from the selected features of both individual models—SA and 4Ch. All variables were standardized by subtracting the mean for each variable and dividing it by its standard deviation (SD) to allow for more meaningful comparisons. A univariable Cox proportional hazards regression was performed to estimate the 1-year mortality prediction of the REVEAL score, CMR measurements, and the MPCA probabilities. For the multivariable analysis, we planned to include the CMR measurements that were identified in previous prognostic studies, namely right ventricular ejection fraction (RVEF), right ventricular end-systolic volume index (RVESVi), right ventricular end-diastolic volume index (RVEDVi), left ventricular end-diastolic volume index (LVEDVi), left ventricular stroke volume index (LVSVi) and pulmonary artery relative area change (PA RAC).^12,21^ Owing to the high correlation between RVESVi and RVEDVi (*r* = 0.89), we only included RVESVi as the stronger predictor in the multivariable analysis. The proportional hazards assumption was confirmed using scaled Schoenfeld residuals. The *c*-index was used to measure the relative goodness of fit between the different regression models. The *c*-index indicates the rate of correct predictions of survival the model makes. We also computed the Akaike information criterion (AIC) for each model. The AIC estimates the rate of incorrect prediction and compares the quality of different models relative to each other while penalizing the models with more variables. While a higher *c*-index indicates a better model fit, a lower AIC value indicates fewer prediction errors.^22^

In addition, the likelihood ratio test was performed to assess if there is a statistically significant difference between the different models and to determine the additive predictive value of the MPCA probabilities. The models compared were the univariable REVEAL score, the REVEAL score combined with prognostic CMR measurements, and finally a multiple variable model including the REVEAL score, CMR measurement, and the MPCA probabilities. Kaplan–Meier curves were analysed to demonstrate the prognostic value of MPCA predictions dividing patients based on the median MPCA value as the threshold. The high and low mortality risk groups were compared using the log-rank (Mantel–Cox) test. The receiver-operating characteristic curve (ROC) and the area under the curve (AUC) were used to estimate the prognostic accuracy of the different MPCA features.

## Results

### Study population characteristics

**Figure 2:**
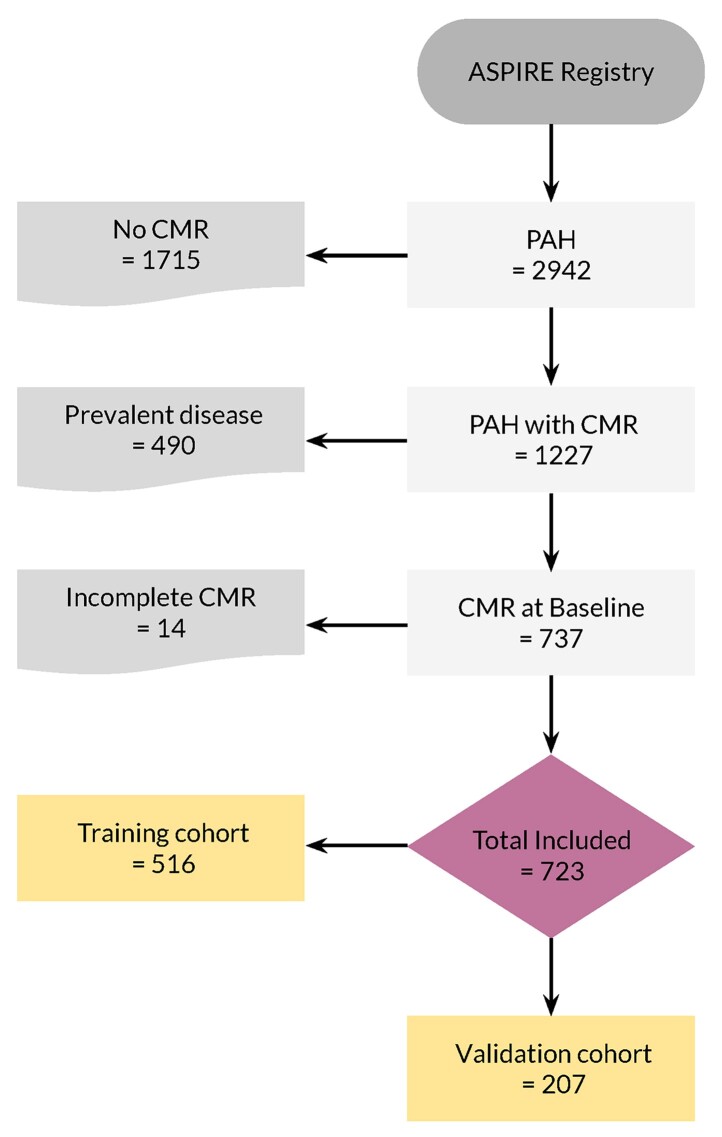
Study participants flow chart.

A total of 737 consecutive incident patients with PAH were identified. Incomplete scans because of claustrophobia or patient intolerance were excluded, leaving 723 scans for the analysis. The training cohort included 516 and the validation cohort 207 subjects (*Figure 2*).

The baseline characteristics of both cohorts are presented in *Table 1*. In summary, the study population were 74% females aged 59 ± 16 years and included PAH secondary to connective tissue disease (CTD) (46%), idiopathic PAH (IPAH) (27%), congenital heart disease (CHD) (16%), secondary to portal hypertension (7%) and other PAH subtypes (4%).

**Table 1:** Baseline characteristics

		Training	Validation	*P*
		N = 516	N = 207	
Age (years)		62 (22)	62 (24)	0.667
Sex (female)		376 (72%)	166 (80%)	0.040
BSA (m^2^)		1.82 ± 0.2	1.83 ± 0.2	0.933
Diagnosis				
	CHD	71 (13%)	34 (16%)	
	CTD	242 (46%)	96 (46%)	
	IPAH	137 (26%)	55 (26%)	
	Portal hypertension	35 (6%)	17 (8%)	
	other PAH	31 (6%)	5 (2%)	
WHO functional Class				0.331
	*I*	2 (0%)	0 (0%)	
	*II*	37 (7%)	11 (5%)	
	*III*	409 (79%)	170 (82%)	
	*IV*	58 (11%)	26 (12%)	
RHC parameters				
			
	*mPAP (mmHg)*	46 (22)	48 (18)	0.164
	*PVR (dyns.s.cm^-5^)*	608 (556)	822 (791)	<0.001
	*PAWP (mmHg)*	11 (5)	10 (4)	<0.001
	RA mean (mmHg)	9 (8)	9 (8)	0.548
	*CO (L/min)*	5 (2)	4 (2)	<0.001
	*SvO_2_ (%)*	66 (13)	66 (14)	0.632
CMR parameters				
	*RVEF (%)*	37 ± 13	36 ± 11	0.539
	*RVESVi (ml/m^2^)*	74 ± 35	76 ± 31	0.122
	*RVEDVi (ml/m^2^)*	113 ± 41	115 ± 39	0.163
	*RVEDMi (g/m^2^)*	27 ± 8	28 ± 8	0.016
	*LVEF (%)*	53 ± 10	53 ± 9	0.966
	*LVESVi (ml/m^2^)*	31 ± 11	31 ± 16	0.212
	*LVEDVi (ml/m^2^)*	67 ± 19	64 ± 21	0.079
	*LVSVi (ml/m^2^)*	36 ± 12	34 ± 9	0.130
	*VMI (ratio)*	0.58 ± 0.2	0.62 ± 0.2	0.010

Data presented as mean ± standard deviation or median (range).

BSA, body surface area; CHD, congenital heart disease; CO, cardiac output; CTD, connective tissue disease; CTEPH, chronic thromboembolic pulmonary hypertension; EDVi, end-diastolic volume index; ESVi, end-systolic volume index; IPAH, idiopathic pulmonary arterial hypertension; LV, left ventricle; mPAP, mean pulmonary artery pressure; PAH, pulmonary arterial hypertension; PAWP, pulmonary arterial wedge pressure; PH, pulmonary hypertension; PVR, pulmonary vascular resistance; RA, right atrium; RHC, right heart catheterization; RV, right ventricle; RVEF, right ventricle ejection fraction; RVEDMi, right ventricular end-diastolic mass index; SV, stroke volume; SvO2 = mixed venous oxygen saturation; VMI, ventricular mass index; WHO, World Health Organization

### Mortality prediction

#### Survival analysis

**Figure 3:**
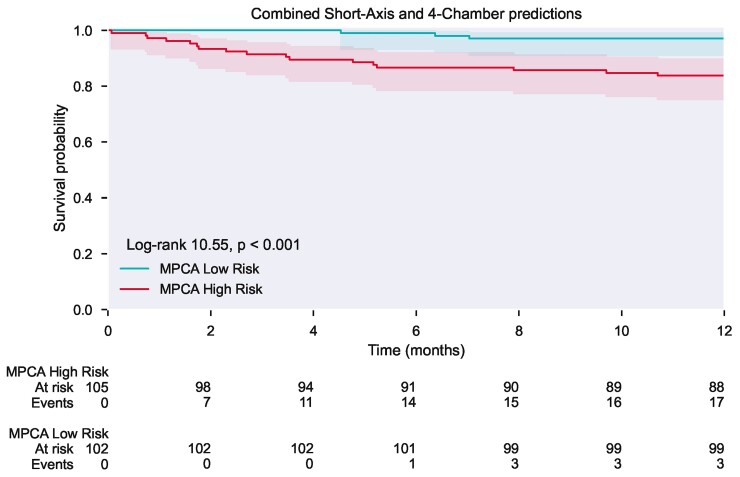
Kaplan–Meier curve. The Kaplan–Meier curve shows the survival of high- and low-risk patients based on the combined short-axis and four-chamber model predictions. The risk threshold was determined based on the median value of the MPCA predictions. The Kaplan–Meier analysis shows a significant difference in survival between the high and low risk of mortality patient groups (log-rank *P* < 0.001).

**Figure 4:**
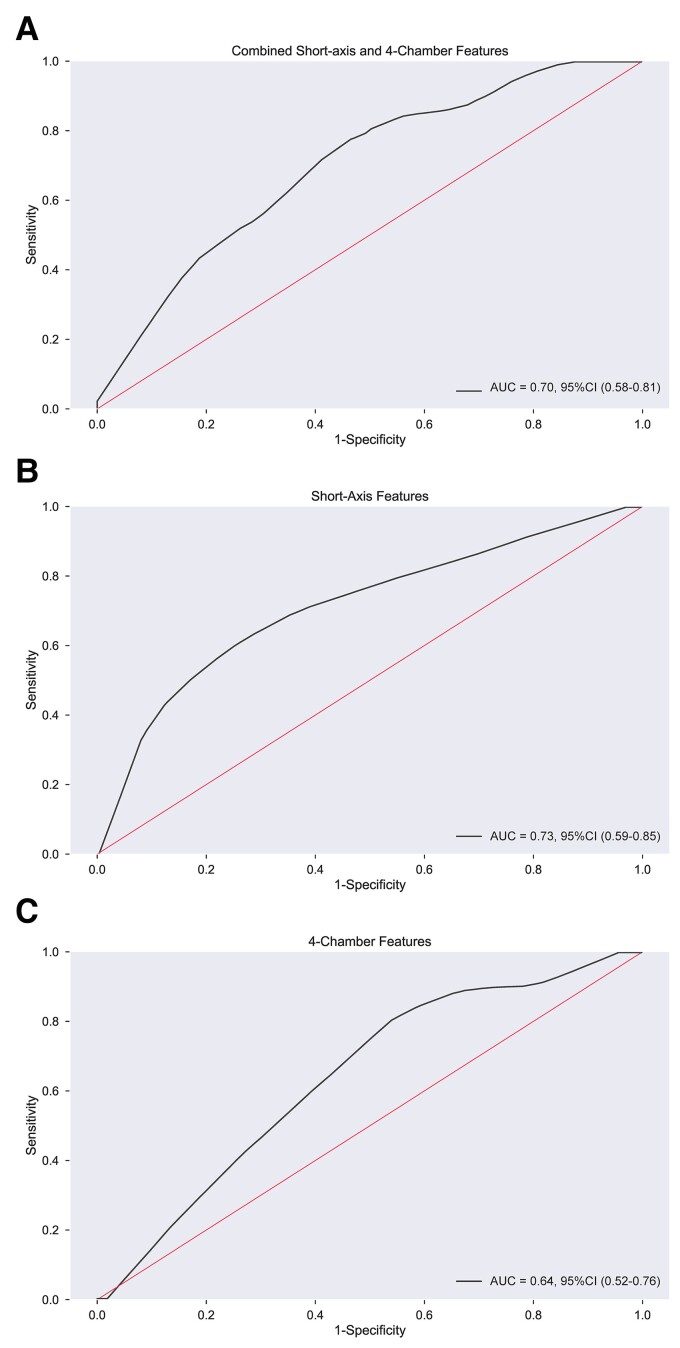
Receiver-operating characteristic curves for 1-year mortality prediction. The prognostic accuracy of the different machine learning models were compared (*A*) combined model, (*B*) short-axis model and (*C*) four-chamber model. The highest area under the curve was achieved with the short-axis model (AUC = 0.73).

The 1-year mortality rate in the validation cohort was 10% with an overall mortality rate over the total follow-up period of 29%. Kaplan–Meier survival analysis demonstrated a significant difference in survival in patients with high and low mortality risk in the validation cohort (log-rank test <0.001) (*Figure 3*). The ROC curve for each model is shown in *Figure 4*. The AUC was 0.73 for the SA model, 0.64 for 4Ch, and 0.70 for the combined MPCA-based features to predict 1-year mortality in the validation cohort.

Univariable Cox regression analysis confirmed a strong prognostic utility of the SA and combined SA and 4Ch MPCA-based predictions (*Table 2*). However, the 4Ch features alone were not significant predictors of mortality. The univariable Cox regression hazard ratios for the demographics, RHC and CMR measurements, functional tests and clinical parameters are shown in *Table 2*. The REVEAL score and, PA RAC and age and sex-adjusted RVESVi were significant predictors of 1-year mortality.

**Table 2:** Univariable Cox proportional hazard regression ratios for 1-year mortality

		HR	95% CI	*P*
Age (years)		1.039	1.005, 1.075	0.**026**
Sex		1.036	0.346, 3.098	0.950
WHO class		2.033	0.781, 5.292	0.146
REVEAL		1.339	1.109, 1.618	**0.002**
RHC parameters				
	*mPAP (mmHg)*	0.982	0.947, 1.017	0.311
	*PVR (dyns.s.cm^-5^)*	1.000	0.999, 1.001	0.616
	*PAWP (mmHg)*	1.012	0.866, 1.183	0.879
	RA mean (mmHg)	1.081	1.020, 1.146	**0.008**
	*CO (L/min)*	0.842	0.599, 1.185	0.324
	*SvO_2_ (%)*	0.969	0.929, 1.009	0.127
CMR parameters				
	*RVEF (% pred)*	0.762	0.445, 1.306	0.324
	*RVESVi (% pred)*	1.699	1.099, 2.628	0.**017**
	*RVEDVi (% pred)*	1.443	0.972, 2.141	0.069
	*RVEDMi (% pred)*	1.113	0.781, 1.587	0.554
	*LVEF (% pred)*	1.275	0.790, 2.060	0.320
	*LVESVi (% pred)*	1.036	0.652, 1.646	0.881
	*LVEDVi (% pred)*	0.918	0.559, 1.509	0.736
	*LVSVi (% pred)*	0.933	0.592, 1.471	0.767
	*VMI (ratio)*	0.962	0.530, 1.748	0.899
	*PA RAC (%)*	0.911	0.838, 0.991	**0.031**
	*Septal angle systole*	0.999	0.972, 1.027	0.941
	*Septal angle diastole*	0.987	0.935, 1.042	0.636
MPCA-based features				
	*SA features*	2.401	1.459, 3.951	**0.001**
	*4-chamber features*	1.472	0.978, 2.216	0.064
	*Combined features*	1.97	1.282, 3.028	**0.002**

CMR parameters are corrected for age and sex (%pred). For abbreviations see Table 1.

#### Additive prognostic value

Several multivariable prognostic models were compared in *Table 3* to compare the predictive value of the REVEAL score alone, REVEAL score combined with CMR measurements or MPCA features and finally REVEAL score combined with CMR measurements and MPCA features. The prognostic models were compared using the *c*-index and AIC test for goodness of fit and the log-rank test to assess the statistical significance of the difference between the models. The univariable REVEAL model allows the assessment of the 1-year risk of mortality based on available composite clinical data alone. Adding the MPCA-based predictions allows evaluating the added incremental value in predicting death compared with REVEAL and segmentation-based CMR parameters.

**Table 3:** *C*-index and Akaike information criterion (AIC) for the univariable and multiple variable Cox regression analysis for the REVEAL score, CMR measurements, and the MPCA machine learning model

	*C*-index	95% CI	AIC	*P*
				Log-rank test
CMR measurements^a^	0.70	0.60–0.80	211	
MPCA^b^	0.70	0.59–0.81	204	
REVEAL score	0.71	0.61–0.81	203	
REVEAL + MPCA	0.76	0.67–0.85	197	**0.003**
REVEAL + CMR measurements	0.78	0.70–0.86	205	**0.003**
REVEAL + CMR measurements + MPCA	0.83	0.76–0.90	193	**<0.001**

A higher *c*-index indicates a better model fit and a lower AIC indicates a relative lower prediction error. The log-rank test indicates that the combination of MPCA, CMR measurements, and REVEAL is statistically significantly more predictive than REVEAL score alone (*c*-index 0.83 vs. 0.72, *P* < 0.001).

a CMR measurements included age and sex corrected right ventricular ejection fraction, right ventricular end-systolic volume index, left ventricular end-diastolic volume index, left ventricular stroke volume index and pulmonary artery relative area change.

b MPCA combined short-axis and four-chamber features

The REVEAL score alone had a *c*-index of 0.71 and AIC of 203. Adding CMR measurements improved the model statistically significantly, to 0.78 and AIC of 205 (log-rank test *P* = 0.003). The model including MPCA prediction, REVEAL score and CMR measurements, showed the strongest prognostic utility (*c*-index: 0.83 and AIC 193, log-rank test *P* ≤ 0.001). The MPCA model alone had similar accuracy to the REVEAL score with a c-index of 0.71 and AIC of 204.

#### Temporal prognostic dynamics

**Figure 5:**
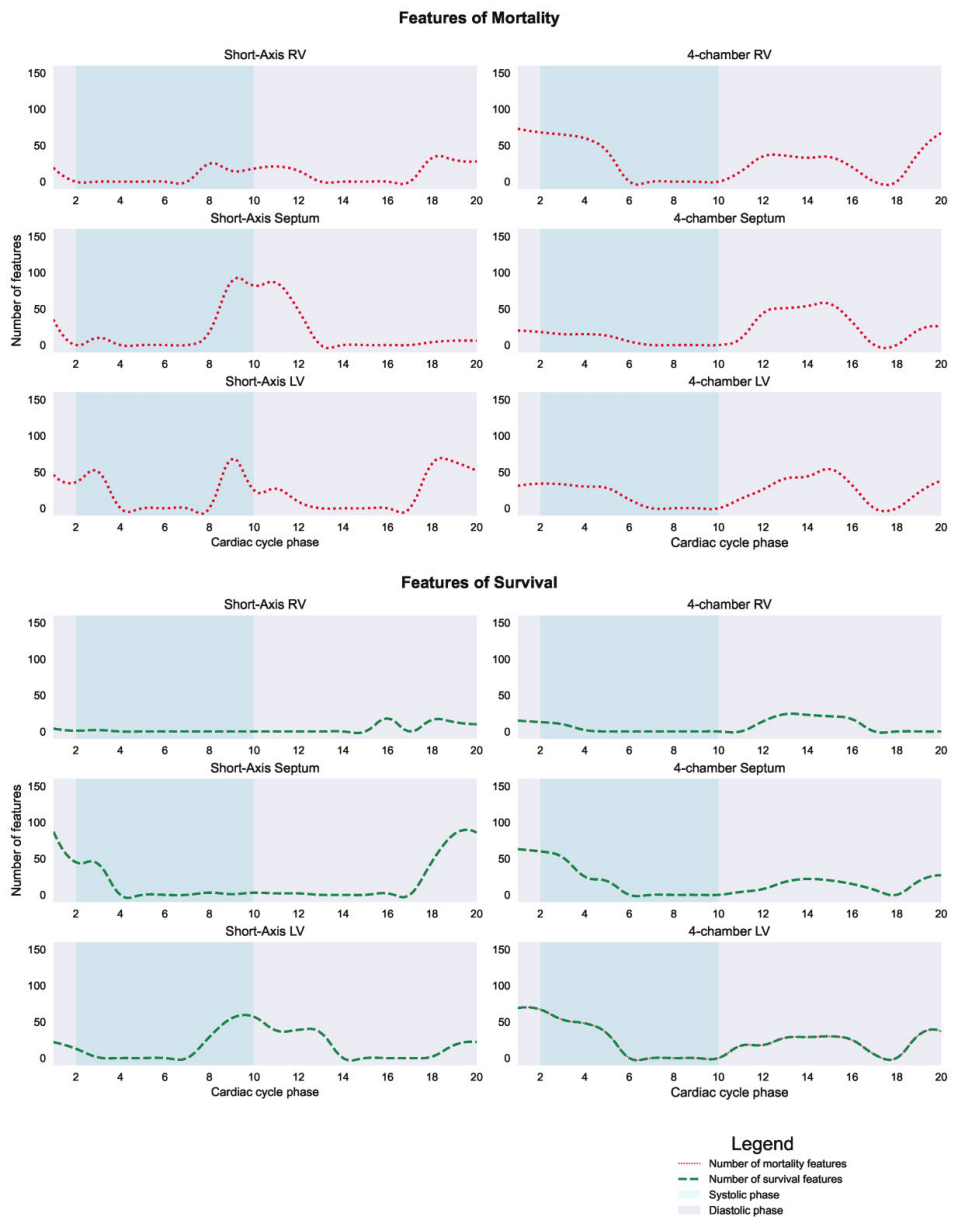
Time-resolved prognostic cardiac features. Features of poor prognosis and also protective features were examined throughout the cardiac cycle on the short-axis and four-chamber views. The most significant cardiac features were the end-systolic and early diastolic septum on both the short-axis and four-chamber views. The RV during systole and LV during diastole also were predictive of 1-year mortality. In contrast, the most important features of survival were the end-diastolic septum on short-axis and four-chamber views and the LV at systole.

**Figure 6:**
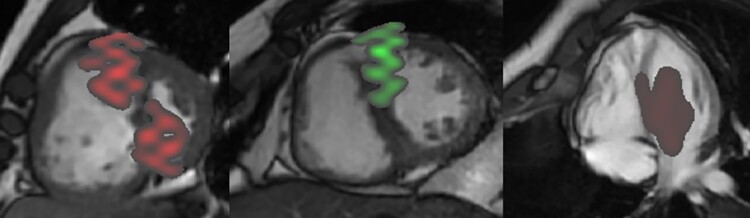
Visualization of prognostic mortality and survival features learnt from the training data. The features were overlaid on three example short-axis/4-chamber images from three different patients with PAH to interpret the corresponding anatomical regions. Left: septum and LV features of high risk of mortality at end-systole. Middle image: Features of survival visualized at the septum at end-diastole. Right: four-chamber view showing high-risk features in the septum and LV in diastole

The MPCA-based features were assessed throughout the cardiac cycle and grouped according to the anatomical region into the right ventricle (RV), left ventricle (LV) and septum. For visualization purposes, we manually segmented the averaged SA and 4Ch slice to group the MPCA features into anatomical regions. The features were divided into low and high-risk features based on the median MPCA feature values used in the Kaplan–Meier analysis (**Figures 5*, *6**). On the SA views, abnormal interventricular septum during systole and particularly at end-systole and the LV chamber during diastole and particularly at end-diastole indicated a higher risk of mortality. On 4Ch views, the features with the highest impact on predicting mortality were at the RV at early systole. A normal LV and interventricular septum in diastole on SA and 4Ch imaging were the strongest predictors of survival, whereas the RV was a poor indicator of survival (*Figure 5*).

## Discussion

This study assessed the prognostic utility of an MPCA-based machine learning model in CMR in patients with treatment-naïve PAH. This is the first study to localize prognostic PAH features with an explainable AI approach dynamically over the cardiac cycle. In addition, we have shown the incremental prognostic value of the MPCA model compared to known prognostic markers such as the REVEAL score and CMR volumetric measurements.

The advantage of using MPCA is its interpretability. The ability to directly relate prognostic features identified in the machine learning process helps understand and explain the machine learning model’s findings. Diagnostic and prognostic models based on deep learning methods have been criticized for creating a ‘black-box’ situation where the predictions are often difficult to comprehend and retrace.^23^ Visualizing the MPCA features throughout the cardiac cycle allowed discerning the most significant discriminatory predictors of death on CMR in PAH. The known prognostic features identified in pulmonary hypertension of diastolic interventricular septal flattening,^24^ reduced LV size and increased RV size^25^ can all be visually assessed on SA images. The most significant features identified in non-survivors on SA imaging were located at the septum at end-systole and LV at end-diastole. Changes in the interventricular septum at end-systole are the result of RV pressure overload. The altered pressure gradient between the LV and RV results in flattening of the septum giving a characteristic D-shaped LV and eventually results in impaired LV diastolic function and reduced LV filling.^26,27^ Survivors showed the opposite with features in the septum at end-diastole and LV at end-systole. We found fewer overall features on SA images at the RV. However, on 4Ch imaging the most significant features were identified in RV systole. Whereas the septal and LV features were less important on 4Ch imaging. The 4Ch view allows assessing the longitudinal RV contractility which for example can be inferred on echocardiogram by assessing the tricuspid annular plane systolic excursion (TAPSE). Right ventricle longitudinal contraction is known to be the larger component of RV contraction and a key prognostic indicator^28–30^ which explains its prognostic importance in PAH.

The MPCA-based model was developed and validated on CMR imaging performed at diagnosis and in treatment-naive PAH patients. Disease severity assessed at baseline assessment is important for planning an optimal treatment strategy. Almost all published prognostic CMR studies in PAH are based on disease prevalent PAH patients in later stages of the disease process.^12^ A meta-analysis of 22 studies and almost 2000 patients with PAH showed that RVEF, RVESVi, RVEDVi, LVEDVi and LVSVi were significant predictors of mortality.^12^ Right ventricle ejection fraction, RVEDVi, LVEDVi, and LVSVi did not predict mortality in our baseline PAH cohort. The MPCA pipeline can therefore elicit cardiac changes before they affect RV function and size and adds prognostic value at baseline evaluation. In addition, comparing the MPCA to the REVEAL score allowed us to evaluate the incremental value benchmarked against a clinically validated baseline prognostic tool. The MPCA-based predictions significantly improved the 1-year mortality prediction of the REVEAL score. The prognostic model accuracy (*c*-index) using REVEAL improved from 71% to 83% (log-rank test *P* < 0.001) when it was combined with CMR data including MPCA predictions and CMR measurements. However, even without REVEAL data, mortality can still be accurately predicted based on MPCA features alone with an accuracy (*c*-index) of 70%.

The application of step-wise cardiac features extraction using CMR has further potential that can be evaluated in future developments. Comparing prognostic features at follow-up with baseline features might provide a better understanding of disease progression on CMR and might offer a standardized disease monitoring tool. In addition, technical improvements would allow to fully automate the prognostic model, which currently requires manual image registration. Deep learning automated landmarking for image registration would reduce the manual processing of CMR images and reduce the time and cost associated with it.^31,32^

### Limitations

This was an exploratory retrospective single centre study on patients with PAH. Findings will need to be confirmed in a prospective trial with an external validation cohort. In addition, applying the model to other diseases and MRI systems would further validate its generalizability.

The MPCA method was applied on cine images of the mid-chamber slice throughout the cardiac cycle. Stack imaging of the whole heart can currently not be included in the MPCA model training. However, because of the strong prognostic signal from the SA and 4Ch cine images we envisage that future developments including 3D data of the heart will further improve prognosis prediction.

## Conclusion

Patient outcome prediction in PAH can be enhanced by adding MPCA-based machine learning to CMR volumetric data and clinical risk scores. The MPCA analysis gives a population insight into the prognostic cardiac features in PAH in an explainable and visualisable approach.

## Supplementary Material

ztac022_Supplementary_Data

## Data Availability

The data underlying this article will be shared on reasonable request to the corresponding author.
